# Structural and biochemical changes underlying a keratoderma-like phenotype in mice lacking suprabasal AP1 transcription factor function

**DOI:** 10.1038/cddis.2015.21

**Published:** 2015-02-19

**Authors:** E A Rorke, G Adhikary, C A Young, R H Rice, P M Elias, D Crumrine, J Meyer, M Blumenberg, R L Eckert

**Affiliations:** 1 Department of Microbiology and Immunology, University of Maryland School of Medicine, Baltimore, MD, USA; 2 Biochemistry and Molecular Biology, University of Maryland School of Medicine, Baltimore, MD, USA; 3 Department of Environmental Toxicology, University of California, Davis, CA, USA; 4 Dermatology Service, Veterans Affairs Medical Center, San Francisco and Department of Dermatology, University of California, San Francisco, CA, USA; 5 The R.O. Perelman Department of Dermatology, Department of Biochemistry and Molecular Pharmacology, New York University Cancer Institute, New York City, NY, USA; 6 Dermatology, University of Maryland School of Medicine, Baltimore, MD, USA; 7 Obstetrics and Gynecology, University of Maryland School of Medicine, Baltimore, MD, USA; 8 Greenebaum Cancer Center University of Maryland School of Medicine, Baltimore, MD, USA

## Abstract

Epidermal keratinocyte differentiation on the body surface is a carefully choreographed process that leads to assembly of a barrier that is essential for life. Perturbation of keratinocyte differentiation leads to disease. Activator protein 1 (AP1) transcription factors are key controllers of this process. We have shown that inhibiting AP1 transcription factor activity in the suprabasal murine epidermis, by expression of dominant-negative c-jun (TAM67), produces a phenotype type that resembles human keratoderma. However, little is understood regarding the structural and molecular changes that drive this phenotype. In the present study we show that TAM67-positive epidermis displays altered cornified envelope, filaggrin-type keratohyalin granule, keratin filament, desmosome formation and lamellar body secretion leading to reduced barrier integrity. To understand the molecular changes underlying this process, we performed proteomic and RNA array analysis. Proteomic study of the corneocyte cross-linked proteome reveals a reduction in incorporation of cutaneous keratins, filaggrin, filaggrin2, late cornified envelope precursor proteins, hair keratins and hair keratin-associated proteins. This is coupled with increased incorporation of desmosome linker, small proline-rich, S100, transglutaminase and inflammation-associated proteins. Incorporation of most cutaneous keratins (Krt1, Krt5 and Krt10) is reduced, but incorporation of hyperproliferation-associated epidermal keratins (Krt6a, Krt6b and Krt16) is increased. RNA array analysis reveals reduced expression of mRNA encoding differentiation-associated cutaneous keratins, hair keratins and associated proteins, late cornified envelope precursors and filaggrin-related proteins; and increased expression of mRNA encoding small proline-rich proteins, protease inhibitors (serpins), S100 proteins, defensins and hyperproliferation-associated keratins. These findings suggest that AP1 factor inactivation in the suprabasal epidermal layers reduces expression of AP1 factor-responsive genes expressed in late differentiation and is associated with a compensatory increase in expression of early differentiation genes.

The epidermis is a highly differentiated structure that acts as a barrier to reduce fluid and nutrient loss, and prevent infection. Assembly of the barrier requires that keratinocytes undergo a complex terminal differentiation process that results in the conversion of proliferating basal cells to suprabasal differentiated keratinocytes. During this process, nuclei, organelles and other cellular structures are destroyed. The ultimate fate of these cells is formation of the stratum corneum, which is comprised of covalently cross-linked proteins, lipids and keratin bundles, and functions as a barrier. Activator protein one (AP1) transcription factors are essential regulators of this process.^1^ These factors form homo- and heterodimers that bind DNA response elements to regulate gene expression.^2^ An example is mutation of a single AP1 transcription factor binding site in the distal regulatory region of the involucrin gene promoter results in a complete loss of involucrin expression in epidermis.^3^ Moreover, selective inactivation of AP1 factor function in epidermis produces phenotypes that mimic human epidermal disease.^4, 5^ To investigate the role of AP1 factors in epidermis, we utilized TAM67, a dominant-negative form of c-jun. TAM67^6^ dimerizes with other AP1 transcription factors. These complexes bind to DNA, but this interaction does not activate transcription and this reduces AP1-mediated gene expression. We targeted TAM67 to the suprabasal epidermis to inhibit AP1 factor-related transcription in this compartment.^4, 5^ Our findings show that TAM67-dependent inactivation of AP1 factor function in the suprabasal epidermis results in increased cell proliferation and delayed differentiation and that this is associated with extensive epidermal hyperkeratosis and formation of constriction rings on the tail and digits^5^ to produce a phenotype that resembles human keratoderma.^4, 5^

However, the structural and biochemical changes underlying this phenotype are not well understood. To assess the biochemical impact of suprabasal AP1 factor inactivation, we analyzed epidermal structure and the corneocyte cross-linked proteome and RNA expression profile. We show that the cornified envelope (CE) cross-linked proteome in AP1 factor-deficient (TAM67-positive) mice is enriched for early envelope precursors, and that late envelope precursors and filaggrin-related proteins are reduced in level. In many cases, these changes are reflected in parallel changes in gene expression. This phenotype is associated with reduced cornified envelope formation, reduced formation of filaggrin-type keratohyalin granules and keratin filaments, and abnormal desmosome formation, lipid processing and desquamation.

## Results

### TAM67-rTA mice develop a unique phenotype

**Figure 1:**
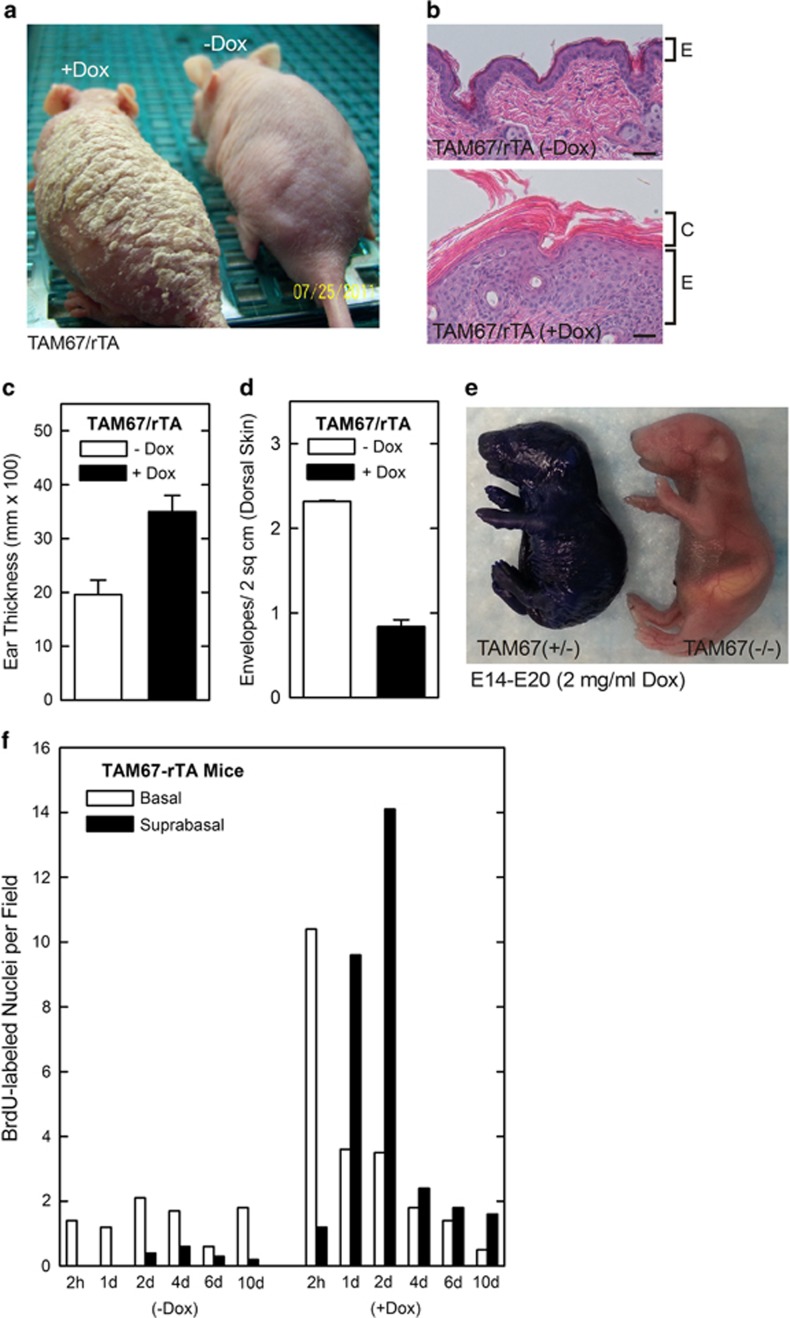
Impact of suprabasal epidermal AP1 factor inactivation on epidermal phenotype . (**a** and **b**) TAM67-rTA mice were treated with 0 or 2 mg/ml doxycycline for 7 days and the mice were photographed and skin sections were processed and stained with hematoxylin and eosin. E indicates the epidermis and C the cornified layers. (**c**) Increased ear thickness in TAM67-FLAG-positive epidermis. Ear thickness was monitored using calipers at 7 days after initiation of doxycycline treatment. (**d**) Reduced cornified envelope number in TAM67–FLAG-positive epidermis. Epidermis scale was collected from TAM67-rTA mice treated as above. The scale was then boiled in detergent and reducing agent and surviving envelope structures were counted. The values are mean±S.E.M. of three separate experiments. Envelope number is significantly reduced (*P*<0.001, *n*=3). (**e**) TAM67 expression compromises barrier function. Pregnant female mice were treated with 2 mg/ml doxycycline beginning on E14 and the embryos were removed and stained with toluidine blue at E20. All TAM67-positive mice displayed compromised barrier function. (**f**) TAM67 expression is associated with increased cell proliferation. Adult TAM67-rTA mice were treated with 0 or 2 mg/ml doxycycline and on day 4 were injected IP with 50 mg of BrdU per kg body weight. At 2 h to 10 days animals were killed and the epidermis was sectioned and stained with anti-BrdU. The number of basal and suprabasal BrdU-positive cells were counted at each time point. Similar findings were observed in each of two experiments

At 2 weeks after induction of TAM67 expression in the suprabasal epidermis, TAM67-rTA mice are covered with scale over the trunk and appendages (Figure 1a).^5^ Microscopic examination reveals that the epidermis is four times thicker than in TAM67-negative mice, and the histology suggests there is enhanced proliferation and cornification (Figure 1b) which is also reflected in increased ear thickness (Figure 1c). However, although extensive hyperkeratosis is evident (Figure 1b), the number of CEs, as counted following boiling in SDS and reducing agent, is reduced (Figure 1d), indicating that the envelopes are not well formed. In addition, there is a 13% reduction in body weight in adult TAM67-positive mice that we believe is caused by increased evaporative water loss due to compromised barrier function. To assess the impact of loss of AP1 factor functional inactivation on barrier integrity, TAM67 expression was induced on E14 and embryos were collected on the day before birth (E20) to assess barrier integrity. Figure 1e shows that the TAM67-expressing littermate has a clear barrier defect as evidenced by enhanced epidermal dye uptake.

To measure changes in keratinocyte proliferation, 8-week-old adult female TAM67-rTA mice were treated with 0 or 2 mg/ml doxycycline for 4 days.^4^ Mice were then injected with bromodeoxyuridine (BrdU) and the fate of the BrdU-labeled cells was assessed by sectioning epidermis and staining with anti-BrdU. Counts of labeled cells at 2 h suggest that seven times more cells are dividing in the TAM67-positive epidermis and that the majority of the cells are in the basal layer (Figure 1f). In control (-Dox) epidermis the number of labeled cells remains relatively stable and most labeled cells localize to the basal layer. In contrast, by 24 and 48 h, the majority of the labeled cells are suprabasal in the TAM67-positive epidermis; moreover, at day 4 the number of labeled cells is reduced, and by 10 days 80% of the labeled cells have been released from the surface. In contrast, in TAM67-negative epidermis only 1% of labeled cells have been released by day 10.

**Figure 2:**
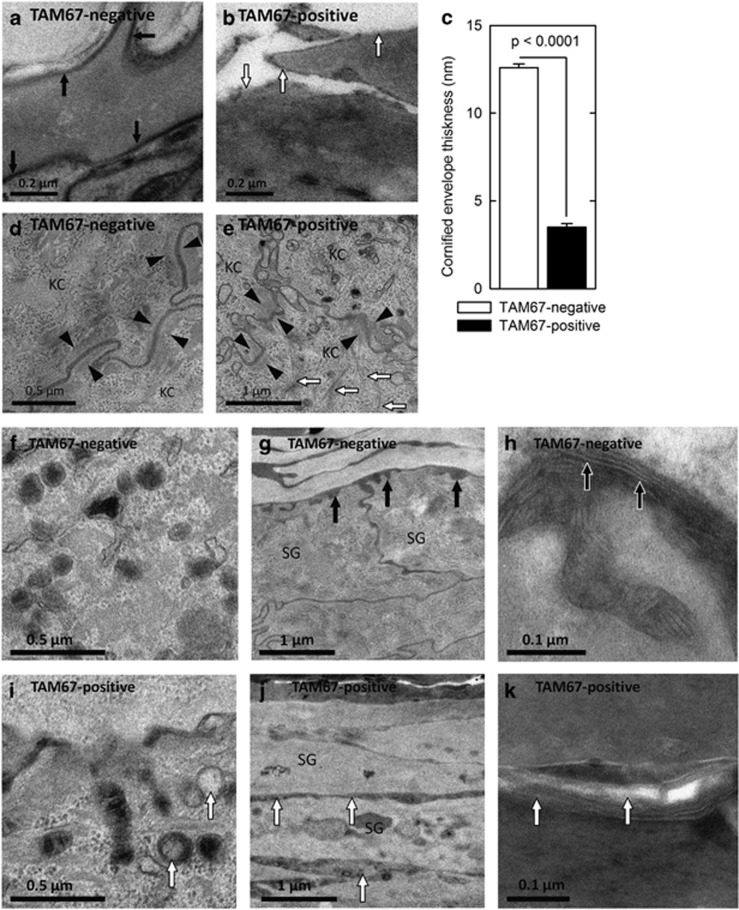
Attenuated cornified envelope, keratin filament and desmosome formation and altered lipid secretion in TAM67-positive epidermis. (**a** and **b**) Electron microscopy reveals uniform cornified envelopes (black arrows) in stratum corneum of TAM67-negative as compared with less-developed envelopes (white arrows) in TAM67-positive epidermis. (**c**) Quantification of the cornified envelope thickness. The values are mean+S.E.M. (**d** and **e**) TAM67-negative epidermis demonstrates compact desmosomes flanked with keratin filaments (black arrowheads) compared with disorganized desmosomes (black arrowheads) and haphazardly arranged keratin filaments (white arrows) in TAM67-positive epidermis. KC indicates keratinocyte. Epidermis from TAM67-negative mice is characterized by lamellar bodies of uniform size filled with stacked membranous lipid contents (**f**), lipid secretion at the SG–SC junction (**g**, black arrows) and processing of secreted lipid into extended membrane arrays (**h**, ruthenium stained, black arrows). In contrast, TAM67-rTA epidermis displays abnormal lamellar body contents (**i**, white arrows), premature lipid secretion in the middle SG layers (**j**, white arrows) and incomplete post-secretory processing of secreted lipid (**k**, white arrows). SG, stratum granulosum; SC, stratum corneum

EM images of TAM67-positive epidermis demonstrate reduced cornified envelope thickness (Figures 2a, b and c), and abnormal desmosomes and keratin filaments (Figures 2d and e). TAM67-positive epidermis exhibits abnormal lamellar body contents (Figures 2f/i), premature lamellar lipid secretion (Figures 2g/j) and incomplete processing of secreted lipid (Figures 2h/k).

### The cross-linked proteome

To understand the biochemical basis for these changes, we examined the composition of epidermal scale from TAM67-positive and -negative epidermis using proteomic methods.^7, 8^ Cornified material was collected and extracted with SDS to remove soluble proteins and fatty acids. The residual was protease digested and analyzed by mass spectrometry.

**Figure 3:**
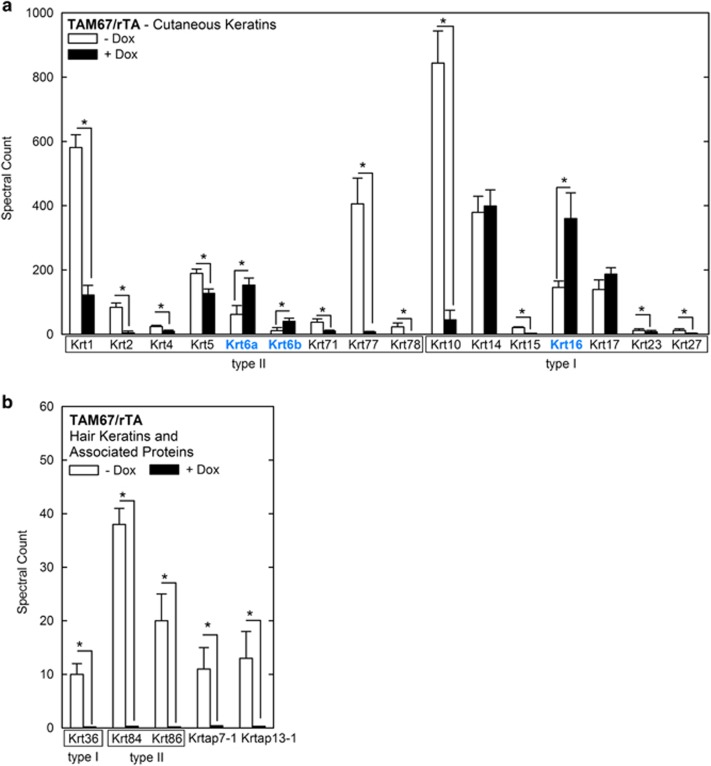
Keratins and keratin-associated protein levels in the cross-linked proteome. (**a** and **b**) Epidermal scale and cornified material were analyzed from three TAM67-positive and three TAM67-negative mice. Samples were processed for mass spectrometry as outlined in Materials and Methods.^8^ The values are weighted spectral counts shown as the mean±S.D.^69^ The asterisks indicate a significant difference (*P*<0.001, *n*=3)

Analysis of keratin composition in TAM67-positive epidermis reveals a decrease in Krt1, 2, 4, 5, 71, 77 and 78 (type II) and Krt10, Krt15, Krt23 and Krt27 (type I; Figure 3a) and an increase in the hyperproliferation-associated keratins, Krt6a, Krt6b and Krt16 (Figure 3a). We also explored the impact on hair keratins and hair keratin-associated proteins. Figure 3b shows that Krt36, 84 and 86 are decreased, as are the Krtap7-1 and Krtap13-1 hair keratin-associated proteins.

**Figure 4:**
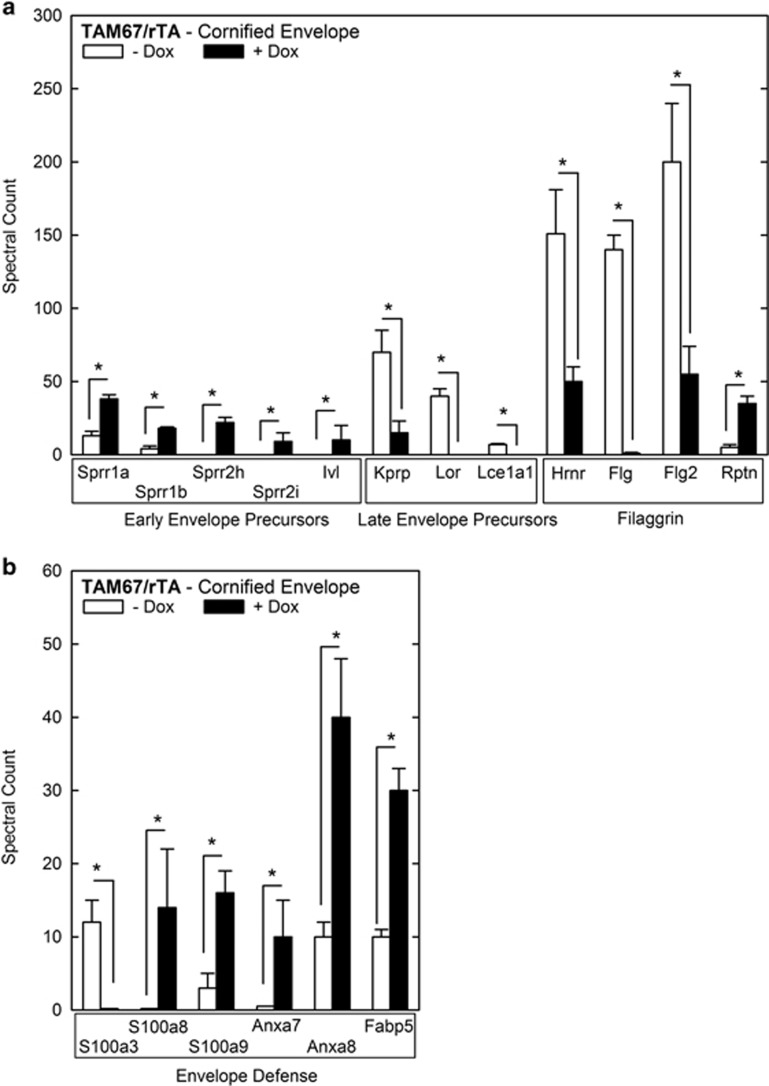
Envelope precursor and defense levels in cross-linked proteome. (**a** and **b**). Level of early envelope, late envelope, filaggrin-related proteins and envelope defense proteins in the cross-linked proteome. Epidermal scale and cornified material was collected and processed as described in the legend to Figure 3. The values are spectral units shown as the mean±S.D. The asterisks indicate a significant difference (*P*<0.001, *n*=3)

We detected significant changes in the level of 12 CE precursors (Figure 4a). Precursors expressed early in normal differentiation are increased in AP1 factor-deficient mice. In contrast, late envelope precursors (keratinocyte proline-rich proteins, loricrin, Lce1a1) are reduced in level, as are most of the S100 fused-type proteins (filaggrin related; Figure 4a). It is interesting that many of these genes are positively regulated by AP1 transcription factors.^9^

Annexin, fatty-acid binding and S100 proteins form functional complexes that are involved in response to pathogen invasion and inflammation.^10, 11, 12^ S100a3 is reduced, but S100a8 and S100a9 are increased (Figure 4b). In addition, the S100 interacting proteins, annexin 7, annexin 8 and Fabp5 are increased. This suggests a tissue inflammatory response.

**Figure 5:**
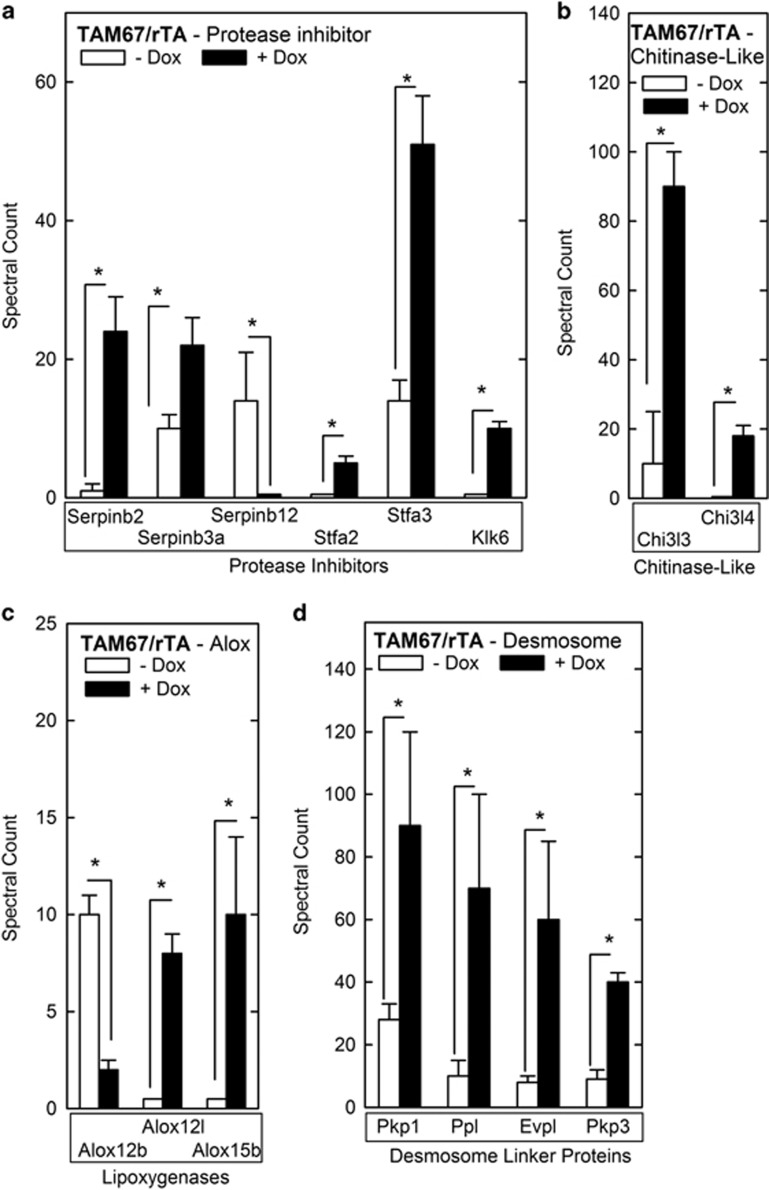
Protease inhibitor, chitinase-like protein, lipoxygenase and desmosome proteins in cornified envelope. (**a**–**d**) Level of the indicated proteins in epidermal scale and cornified material collected and processed as described in the legend to Figure 3. The values are spectral units shown as the mean±S.D. The asterisks indicate a significant difference (*P*<0.001, *n*=3)

Proteolysis is an important event in the epidermis.^13^ Composition analysis reveals enhanced serpin and stefin levels (Figure 5a). Serpins are irreversible serine protease inhibitors^14, 15^ and serpin2 and serpin3a are increased in cross-linked material from TAM67-expressing epidermis, while serpinb12 level is reduced. Stefin 2 and 3 (Stfa2 and Stfa3) are members of the cystatin family and inhibit lysosomal cysteine proteinases including cathepsin B, H, K, L and S.^16, 17^ Stfa2 and Stfa3 are increased in cross-linked material from TAM67-expressing epidermis. In addition, kallikrein-related peptidase 6 (Klk6), which has trypsin-like properties, is elevated in level (Figure 5a).

Chitinases hydrolyze chitin.^18^ Mice express two catalytically inactive chitinase-like genes, Chi3l3 (Ym1) and Chi3l4 (Ym2).^19^ Increased Chi3l3 and Chi3l4 are observed in the epidermis of mouse models of chronic proliferative dermatitis and are derived from macrophages, dendritic cells and mast cells.^19^ We observed increased Chi3l3 and Chi3l4 content in the CE of TAM67-expressing mice (Figure 5b). 12/15-Lipoxygenases catalyze the oxidation of free and esterified fatty acids to generate bioactive lipids that form ceramides, which are required for formation of the lipid barrier.^20^ Alox12b, Alox12l and Alox15b, are murine lipoxygenase genes,^20^ and these proteins are increased in TAM67-expressing epidermis (Figure 5c).

Desmosomes are essential barrier components,^13^ and are altered in TAM67-positive epidermis (Figure 2e). The desmosome includes membrane-spanning core proteins, including desmocollins (DSCs) and desmoglein (DSGs),^21^ and linker protein components, including envoplakin (Evpl), periplakin (Ppl), plakophilin 1 (Pkp1) and plakophilin 3 (Pkp3), which tether these core proteins to the actin and keratin intermediate filaments.^22^ Analysis reveals no change in the level of desmosomal core proteins in TAM67-positive cross-linked protein preparations; however, there is substantial increase in the level of tethering proteins (Pkp1, Ppl, Evpl and Pkp3; Figure 5d).

### RNA expression

We next used gene array to compare mRNA expression (Table 1). TAM67-negative epidermis, which differentiates normally, is enriched in mRNA encoding cutaneous keratins, hair keratins and hair keratin associated proteins, and expression of these genes is reduced in the epidermis of TAM67-expressing epidermis. Cornified envelope precursors also change. Sprr4, an early-expressed envelope marker, and a host of late envelope precursor genes, including loricrin and the late cornified envelope (*Lce*) genes, are enriched in TAM67-negative epidermis. Filaggrin-related proteins, including filaggrin2, trichohyalin and cornulin, are also enriched in TAM67-negative *versus* -positive epidermis.

In contrast, TAM67-positive mice display increased expression of mRNA encoding hyperproliferation-associated keratins (Krt6a, Krt16 and Krt17), early envelope precursors (Sprr2b, Sprr2d, Sprr2e) and type III transglutaminase. We also observed a marked enrichment of mRNA encoding serine protease inhibitors (Serpins, Spink12), proteins involved in inflammation/bacterial defense (S100 proteins, defensins) and chitinase-3-like protein 4 (Chi3l4). We also observed an impact on desmosomal protein expression. DSC 2 and DSG 3, which constitute the core of the desmosome,^21^ were increased.

### Time course of change in expression

**Figure 6:**
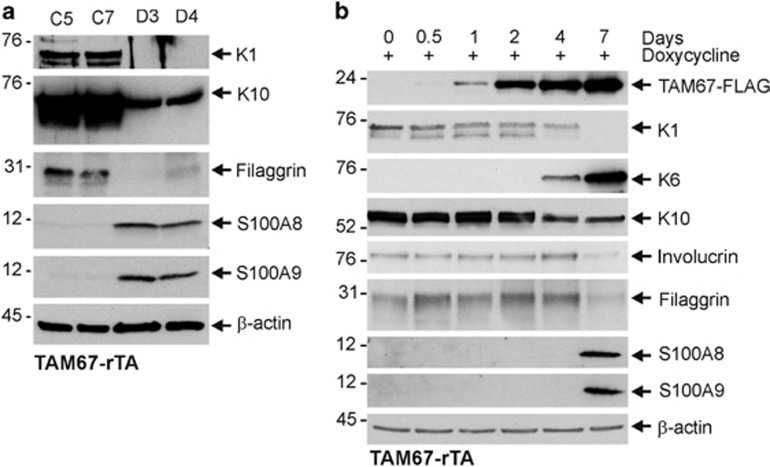
Protein levels in TAM67-negative *versus* -positive epidermis. (**a**) Suprabasal AP1 factor inactivation alters epidermal protein level. TAM67-rTA mice were treated for 7 days with 0 (C5, C7) or 2 (D3, D4) mg/ml doxycycline administered in drinking water. The epidermis was then separated from the dermis using dispase and equivalent protein amounts were electrophoresed on denaturing and reducing 10% polyacrylamide gel. The proteins were transferred to nitrocellulose for incubation with appropriate antibodies.^70^ (**b**) Protein level as a function of time after AP1 factor inactivation. TAM67-rTA mice were treated for 0–7 days with 0 or 2 mg/ml doxycycline administered in drinking water. The level of the indicated proteins was then detected by immunoblot. Similar results were observed in each of three experiments

We next assayed selected proteins to confirm expression changes. Figure 6a compares protein level in epidermal extracts prepared from TAM67-negative (C5, C7) and positive (D3, D4) mice. This reveals reduced levels of Krt1 and Krt10 and filaggrin, and increased S100A8 and S100A9 in TAM67-positive epidermis. Time course studies (Figure 6b) reveal TAM67-FLAG expression at 1 day after addition of doxycycline. Krt1, Krt10, involucrin and filaggrin levels are reduced and Krt6 and S100A8 and S100A9 levels increased at days 4 and 7.

## Discussion

We are interested in control of epidermal gene expression by the AP1 family of transcriptional regulators.^23^ This family includes c-jun, junB, junD, c-fos, Fra-1 and Fra-2.^1^ We recently showed that inactivation of suprabasal AP1 factor function in epidermis causes a phenotype characterized by hyperplasia, hyperkeratosis, parakeratosis and pseudoainhum that resembles human keratoderma.^5^ The present study expands on these findings and examines the impact of AP1 factor inactivation on the structure and composition of the CE, and on epidermal keratinocyte gene expression. We show that the keratoderma-like phenotype is associated with defective CE formation. Although the epidermis is thicker and the cornified layer is expanded,^5^ the number of mature envelopes per unit area of skin is markedly reduced, and barrier function is compromised. In addition, electron microscopic examination of the epidermis reveals reduced CE formation, as well as abnormalities at each step of lipid barrier formation: lamellar body loading, lipid secretion and secreted lipid processing. Defective lipid barrier formation likely contributes to the defective skin barrier in these mice and may induce expression of hyperproliferative proteins including Krt6 and Krt16. The premature lipid secretion observed in TAM67-positive epidermis (e.g., in the lower layers of stratum granulosum rather than chiefly at the stratum granulosum-stratum corneum) is also seen in Par2 knockout mice^24^ and other models of abnormal cornification, and may result from reduced entombment of lamellar bodies in the setting of the delayed apoptosis that occurs with epidermal hyperproliferation.

### Keratins, envelope precursors and desmosomes

The keratoderma-like phenotype we observe is associated with reduced expression of differentiation-associated cutaneous keratins, and increased expression of hyperproliferation-associated (Krt6a, Krt16 and Krt17) keratins. Analysis of the cross-linked proteome in these mice reveals a reduction in differentiation-associated type I and type II cutaneous keratins (Krt1, Krt2, etc.) coupled with increased levels of Krt6a, Krt6b and Krt16. First, this is consistent with the likelihood that keratins which are available during CE formation are used in proportion to their abundance in keratinocytes. Second, these finding are consistent with reduced keratinocyte differentiation. Third, it appears that the change in keratin composition is associated with altered keratin filament formation, as the EM images suggest a decrease in keratin filaments in TAM67-positive epidermis. This may also have to do with the substantial reduction in the level of keratin organizing proteins, such as filaggrin.

Interestingly, we also observe a dramatic reduction in hair keratin and hair keratin-associated protein level in cross-linked proteome preparations derived from TAM67-positive epidermis (Figure 3b). Expression of a large number of genes encoding this class of proteins is reduced in TAM67-positive epidermis (Table 1). The hINV promoter,^23^ which we used to target TAM67 to epidermis, is expressed in the hair follicle.^25^ This suggests that hair follicle-localized AP1 factor function is required for expression of these hair follicle keratins.

We also observe increased expression of early CE (sprr proteins and involucrin) and reduced expression of late CE (Kprp, Lor, Lce1a1) precursors and filaggrin-related proteins (hornerin, filaggrin, filaggrin2). Many *Lce* genes are reduced in expression, as are genes encoding loricrin, filaggrin2, trichohyalin and cornulin. Here again, this finding suggests a key role for AP1 transcription factors in maintaining appropriate expression. Considering the marked reduction in filaggrin level, it is interesting that the level of bleomycin hydrolase, a neutral cysteine protease that breaks down filaggrin into amino acids,^26^ is not altered in expression or in cross-linked proteome content (not shown).

Desmosome protein expression is also altered. Desmoplakin is an abundant desmosomal protein and epidermal genetic disorders are associated with mutation of the desmoplakin gene.^27^ Desmoplakin haploinsufficiency is associated with striate palmoplantar keratoderma^27, 28^ and a desmoplakin mutant causes autosomal recessive cardiomyopathy-associated PPK.^28^ Plakoglobin mutations are associated with autosomal recessive Naxos Syndrome^29^ and plakoglobin null mice display a severe skin phenotype.^30^ DSCs and DSGs are core components to the desmosome that are expressed in epidermis. DSG1 and DSC1 are expressed in the suprabasal, whereas DSG2, DSG3 and DSC2 are expressed in the basal epidermis.^29^ Consistent with reduced differentiation/enhanced proliferation of TAM67-positive epidermis, the basal layer DSCs, Dsc2 and Dsg3, are increased in expression. It is interesting that the cornified layer has abnormal desmosomes. In this context, we note that caspase 14, which localizes at desmosomes in epidermis,^31^ and may impact desmosome processing, is markedly reduced in preparations derived from TAM67-expressing epidermis (not shown). In addition, cathepsin D that degrades desmosomes during desquamation,^32^ is 50% reduced in TAM67-positive envelopes (not shown). Moreover, the level of mRNA encoding the hair follicle DSG, Dsg4,^29^ is reduced in TAM67-positive epidermis (not shown). These findings are consistent with a general reduction in expression of hair follicle associated genes (e.g., hair keratins and associated proteins).

As loss of suprabasal AP1 factor function results in loss of differentiation-associated keratin, envelope precursor and desmosomal protein expression, it is not surprising that the epidermis, as shown in Figures 1 and 2, is visually, histologically and functionally impaired. To better understand this, we monitored markers associated with epidermal response to stress.

### Epidermal defense

S100 proteins and annexins interact to from macromolecular complexes^33, 34^ that are incorporated as components of the CE where they have inflammatory and bacterial defense actions.^12, 34^ S100A8 and S100A9, which are markedly enriched in TAM67-positive epidermis, are frequently highly expressed in hyperproliferative diseases.^35^ S100A8 and S100A9 mRNA levels are among the most increased in TAM67 epidermis. Expression of two defensin genes, *Defb3* and *Defb4*, is also markedly increased. Taken together, these results suggest a response to impending or active infection.

### Lipoxygenases

Lipoxygenases are a class of ubiquitous nonheme iron containing dioxygenases that catalyze the stereo- and regiospecific incorporation of molecular oxygen into polyunsaturated fatty acids containing a (*cis*,*cis*)-1,4-pentadiene structure. The products are hydroperoxides that are rapidly transformed into other compounds.^36^ The mouse genome encodes seven lipoxygenase genes on mouse chromosome 11. The six human genes are on chromosome 17.^37^ Lipoxygenase genes are classified based on the positional specificity of arachidonic acid oxygenation (e.g., 5-, 8-, 12- and 15-LOX) and the tissue of discovery (i.e. platelet-type 12-LOX (p12-LOX)). Our analysis reveals that lipoxygenease gene expression and lipoxygenase incorporation into CEs is altered in TAM67-positive epidermis and suggests these changes may contribute to development of the abnormal epidermal phenotype.

The *ALOX12B* gene (ID 11686) product catalyzes oxidation of acyl-ceramide to form ox-acyl-ceramide during synthesis of the cornified lipid envelope.^38^ Our studies show that Alox12b levels are reduced in the cross-linked proteome of TAM67-positive epidermis. Mutations that inactivate the *ALOX12B* gene product are associated with autosomal recessive congenital ichthyosis (ARCI) types including harlequin ichthyosis, lamellar ichthyosis and congenital ichthyosiform erythroderma.^39^ These rare diseases are characterized by epidermal scaling and birth as collodion babies.^40^ After loss of the collodion membrane in the first weeks of life these patients exhibit generalized scaling, erythema, epidermal hyperplasia and hyperkeratosis. It is likely that this phenotype is a compensatory response to the loss of cutaneous barrier function. These patients may also display palmoplantar hyperlinearity with or without keratoderma. A recent study identified mutations in ALOX12B as a cause of a form of ARCI called ‘self-healing collodion baby'.^41^ A specific ALOX12B (p.Tyr521Cys) mutation is frequently present in these patients and small deletion, insertion and single-nucleotide mutations are also observed.^39, 41, 42^ Mice harboring a loss-of-function Alox12b mutation display an ARCI phenotype,^43^ and Alox12b knockout mice are born with red/shiny skin and rapidly desiccate and perish after birth.^44^ Our studies showing reduced Alox12b level in CE are consistent with reduced ceramide processing and reduced assembly of the cornified lipid envelope as a contributing factor in phenotype development. This is consistent with our EM micrographs showing a loss of lipid envelope formation.

The *Alox15b* gene (ID 11688) product converts 8(S)-hydroperoxyeicosatetraenoic acid (8S-HPETE) from arachidonic acid and also oxygenates *α*-linolenic acid and docosahexaenoic acid and converts 5-HPETE to LTA4.^45^ Alox15b is expressed in cutaneous adnexa and hair follicles, and is also abundant in sebaceous, eccrine and apocrine glands.^46^ The presence of ALOX15B in secretory glands suggests a role in regulating secretory processes.^46^ Alox15b is also expressed in mouse and human interfollicular epidermis. In mouse epidermis, expression is observed in the differentiated layers.^47^ Alox15b enhances keratinocyte differentiation and suppresses cell growth.^48^ Expression of Alox15b in cultured cells suppresses cell growth via effects on p38 mitogen-activated protein kinase signaling.^49, 50^ Similarly, transgenic mice overexpressing Alox15b display enhanced epidermal differentiation, and reduced tumor formation.^48, 50^ Likely as a compensatory response, Alox15b level is substantially increased in the granulosum of I*κ*B*α*-deficient mice, which display a psoriatic phenotype.^51^ Alox15b is also induced following treatment of epidermis with phorbol ester.^47^ Thus, it has been proposed that Alox15b is a differentiation marker and may be involved in promoting terminal differentiation. We observed a substantial increase in Alox15b level in the cross-linked proteome prepared from TAM67-positive epidermis. We propose that this is a compensatory response as part of a program to restore normal differentiation.

The *Alox12l* gene (ID 11687) encodes leukocyte-type 12-lipoxygenase, which is produced by peritoneal macrophages, but is not produced in epidermis.^37^ Our finding that Alox12l is elevated in the CE in TAM67-positive mouse epidermis probably represents macrophage invasion into the epidermis. Finally, we observed a substantial increase in the level of mRNA encoded by the *Alox12e* gene (ID 11685). This lipoxygenase is predominantly expressed in the suprabasal epidermis and part of the hair follicle.^52^ We are not sure why this gene is increased in expression, except that it may be a compensatory mechanism associated with reduced differentiation.

### Chitinase-like proteins

Chitinases are enzymes that breakdown chitin. Two active enzymes have been identified and several related proteins that lack enzymatic activity.^19^ It has been proposed that active chitinases are involved in defense in response to infection; however, the function of the inactive (chitin like) enzymes is not well understood. The level of these proteins is markedly increased in the chronic proliferative dermatitis mouse. This is associated with epidermal thickening, dermal neovascularization and fibrosis, and infiltration by eosinophils, macrophages and dendritic cells.^53, 54^ Chi3l3 and Chi3l4 accumulation in epidermis is localized to macrophages, dendritic cells and mast cells.^19^ This may suggest that the increase in these proteins in TAM67-expressing epidermis is owing to immune cell invasion.

### Summary

Previous studies suggest that AP1 factors are important regulators of differentiation in the human epidermis.^1, 2, 55^ The present study expands the number of potential genes that may be regulated by AP1 factors. Moreover, altered expression of these genes is associated with other skin diseases. Some of these genes are candidates as direct targets of AP1 factor action (e.g., late envelope precursors, filaggrin, loricrin, etc.),^9, 56, 57, 58, 59, 60, 61^ whereas other genes likely change expression and envelope incorporation as a compensatory response to reduced differentiation or loss of barrier function. Additional studies will be necessary to sort out the regulatory arrangements. An important point is that TAM67-associated AP1 factor inactivation in the suprabasal epidermis produces a dramatic phenotype^4, 5^ (Figure 1), but TAM67 expression in the basal epidermis does not.^62^ This supports our contention that perturbing transcription factor function can produce changes in phenotype in an epidermal localization-specific manner.^4^

## Materials and Methods

### Animals

The TAM67-rTA mice used in this study are the offspring of a cross of hINV-rTA +/− mice and TetO-TAM67-FLAG mice and are maintained in a hairless SKH1 background.^5^ The hINV-rTA mice express the reverse-tetracycline activator (rTA) protein targeted to the suprabasal epidermis. Interaction with doxycycline converts the rTA protein to an active conformation that binds to the TetO in the TetO-TAM67-FLAG cassette to drive TAM67 expression. Thus, treatment of TAM67-rTA mice with doxycycline in the drinking water results in suprabasal TAM67-FLAG expression.^4, 5^ The transgenes are maintained in the SKH-1 hairless genetic background to facilitate visualization of the epidermal phenotype.^5^ Mice were housed in the University of Maryland School of Medicine animal facility in compliance with NIH regulations. Eight-week-old female TAM67-rTA mice were treated with 0 or 2 mg/ml doxycycline in their drinking water for 0–2 weeks to induce the phenotype. At various time points, the mice were sedated, euthanized and the dorsal skin and epidermis were collected.

### Barrier integrity, envelope counting and BrdU labeling assays

To assess the impact of loss of AP1 factor function on barrier integrity, a TetO-TAM67-FLAG male was bred with a hINV-rTA +/− female. At E14, the pregnant female was treated with 2 mg/ml doxycycline in the drinking water and embryos were collected on the day before birth (E20). Pregnant females were anesthetized and killed by cervical dislocation and the embryos were removed, washed with phosphate-buffered saline and treated sequentially with 25, 50, 75 and 100% methanol. The mice were washed with phosphate-buffered saline and incubated for 10 min in a 0.1% toluidine blue solution. The pups were thoroughly washed with phosphate-buffered saline and photographed.

To count cornified envelopes, epidermis from dorsal skin (2 cm^2^) was collected, minced and boiled in 2% SDS containing 5 mM *β*−mercaptoethanol. Under these conditions, cells are solubilized but CEs survive. An aliquot of the samples was diluted 1 : 10 in sterile water and counted.^33^

For BrdU labeling of epidermis, eight week old female TAM67-rTA SKH1 mice were intraperitoneally injected with 50 mg BrdU per kg body weight. At 0–10 days thereafter, mice were collected and paraffin-embedded/formalin-fixed epidermal sections were prepared and stained with mouse monoclonal anti-BrdU clone BU-33 (Sigma, B8434, St. Louis, MO, USA). Primary antibody binding was visualized using biotin-labeled anti-mouse IgG in the Mouse on Mouse detection kit from Vector Labs (Burlingame, CA, USA) (PK-2200).

### Electron microscopy

Skin biopsies were taken for electron microscopy.^63^ Briefly, samples were minced to <0.5 mm^3^, fixed in modified Karnovsky's fixative overnight and postfixed in 1% aqueous osmium tetroxide containing 1.5% potassium ferrocyanide or in 0.2% ruthenium tetroxide (the latter only where indicated in figure legends). After fixation, all samples were dehydrated in a graded ethanol series and embedded in an Epon-epoxy mixture. Ultrathin sections were examined, with or without further contrasting with lead citrate, in a JEOL electron microscope (JEOL USA, Inc., Peabody, MA, USA), operated at 60 kV. Cornified envelope thickness was determined by three independent measurements of each envelope present on three fields for each genotype using Gatan Imaging software (Pleasanton, CA, USA).

### Immunoblot protein detection

Equivalent amounts of protein were electrophoresed on denaturing and reducing polyacrylamide gels and transferred to nitrocellulose. The nitrocellulose membrane was blocked by 5% nonfat dry milk for 1 hour and incubated with primary antibody (diluted 1 : 1000) in 5% nonfat dry milk followed by incubation with secondary antibody (diluted 1 : 5000) for 2 h at 37 °C. Secondary antibody binding was visualized with ECL Prime (Amersham) chemiluminescence detection reagent.

### Mass spectrometry sample preparation

After the outer scale was removed and collected by scraping, the remaining epidermis was collected from the dermis by heating the excised skin in water at 52 °C for 1 min.^64^ The samples were then quick frozen at −70 °C. Scale samples were heated in 2% SDS −0.1 M sodium phosphate (pH 7.8) for 10 min in a 90 °C water bath and centrifuged for 5 min at 20 000 × g. Extraction of corneocytes with sodium dodecyl sulfate (SDS) yields proteins linked by disulfide and isopeptide bonding, nearly all of which are represented in the isopeptide cross-linked (envelope) fraction.^8, 33^ The cloudy supernatant was discarded, and the samples were washed four times with and then resuspended in 2% SDS −0.1 M sodium phosphate, pH 7.8. Samples (0.4 ml) were then reduced with 25 mM dithioerythritol and alkylated with 50 mM iodoacetamide at room temperature in the dark with stirring. The protein components were collected by addition of 1.1 ml ethanol followed by centrifugation, rinsed twice with 67% ethanol and once in 0.1 M ammonium bicarbonate and digested with reductively methylated bovine trypsin as previously described.^8^ The samples were then submitted for mass spectrometry.

### Mass spectrometry

Sample digests (adjusted to equal peptide amounts by A^280^) were acidified with trifluoroacetic acid and directly loaded onto a nano LC column from which peptides were eluted with a formic acid-acetonitrile gradient and analyzed as previously described.^65^ The extraction of peptide spectra was performed using X! Tandem (The GPM, thegpm.org; version CYCLONE (2013.02.01.1)) to search the Uniprot mouse database (86 070 entries) and a database of reverse sequences for estimation of false discovery rate for trypsin digests. Peptide and protein (minimum of two peptides) identifications (0.2 and 3.6% false discovery rates, respectively) were verified by Scaffold version_4.2.1 (Proteome Software Inc., Portland, OR). Numbers of distributed spectral counts (called weighed spectral counts in Scaffold) were tabulated using experiment-wide grouping, which provides more accuracy than total spectral counts for proteins (e.g., certain keratins) with shared peptides.^65^

### Gene array analysis

For gene array analysis, 8-week-old female TAM67-rTA SKH1 mice were treated for 8 days with 0 or 2 mg/ml doxycycline and the epidermis was recovered using dispase.^4, 5^ Approximately 5–8 *μ*g of total RNA was reverse transcribed, amplified and labeled as described.^66^ Labeled cRNAs were hybridized to Affymetrix Mouse Gene 1.0 ST Arrays, which cover 28 853 genes. The arrays were washed, stained with anti-biotin streptavidin-phycoerythrin labeled antibody using fluidics station and then washed again according to the Affymetrix protocol. Data analysis was described previously.^67^ Briefly, intensity values were obtained using Microarray Suite version 5.0 (Affymetrix, Santa Clara, CA, USA), and scaled by calculating the overall signal for each array. Differential expression was defined as a twofold or greater difference in expression. For annotation we used the DAVID program [http://david.abcc.ncifcrf.gov].^68^ DAVID provides tables containing functional and ontological details of the genes in the uploaded list, charts containing ontological categories, pathways and so on, over-represented in the gene lists, as well as clusters of such ontological categories, which consolidates redundancies and overlaps, transcription factors over-represented in the promoters of the genes, as well as sublists of genes specific for each ontological category.

## Figures and Tables

**Table 1:** Gene array analysis

**Ratio: (log2)**^a^ ***TAM67+/TAM67−***	**Gene name**	**Entrez gene ID**	**Description**	**Category**
*Gene expression elevated in TAM67-negative epidermis*				
				Cutaneous keratins
−4.08	*Krt71*	56735	Keratin 71	
−3.64	*Krt25*	70810	Keratin 25	
−3.47	*Krt27*	16675	Keratin 27	
−3.47	*Krt26*	320864	Keratin 26	
−3.04	*Krt15*	16665	Keratin 15	
−3.02	*Krt28*	70843	Keratin 28	
−2.88	*Krt72*	105866	Keratin 72	
−2.41	*Krt77*	406220	Keratin 77	
−2.37	*Krt73*	406220	Keratin 73	
−2.32	*Krt23*	94179	Keratin 23	
−2.13	*Krt78*	332131	Keratin 78	
				Hair keratins and associated proteins
−3.66	*Krt33a*	71888	Keratin 33a	
−3.19	*Krt86*		Keratin 86	
−2.47	*Krt31*	16660	Keratin 31	
				
−4.43	*Krtap7-1*	71363	Keratin associated protein 7-1	
−4.39	*Krtap8-1*	16703	Keratin associated protein 8-1	
−3.46	*Krtap3-3*	66380	Keratin associated protein 3-3	
−3.27	*Krtap1-5*	69664	Keratin associated protein 1-5	
−3.04	*Krtap6-1*	100040214	Keratin associated protein 6-1	
−3.03	*Krtap16-8*	68484	Keratin associated protein 16-8	
−2.92	*Krtap4-16*	435285	Keratin associated protein 4-16	
−2.91	*Krtap4-1*	665891	Keratin associated protein 4-1	
−2.77	*Krtap8-2*	16704	Keratin associated protein 8-2	
−2.67	*Krtap13-1*	268905	Keratin associated protein 13-1	
−2.64	*Krtap6-3*		Keratin associated protein 6-3	
−2.57	*Krtap4-7*	76444	Keratin associated protein 4-7	
−2.23	*Krtap1-4*	629873	Keratin associated protein 1-4	
−2.03	*Krtap4-2*	68673	Keratin associated protein 4-2	
−1.83	*Krtap3-1*	69473	Keratin associated protein 3-1	
−1.83	*Krtap16-7*	170656	Keratin associated protein 16-7	
−1.80	*Krtap3-2*	66380	Keratin associated protein 3-2	
−1.77	*Krtap1-3*	435273	Keratin associated protein 1-3	
−1.70	*Krtap16-5*	77918	Keratin associated protein 16-5	
				Early envelope
−1.8	*Sprr4*	229562	Small protein-rich protein 4	
				Late envelope
−3.97	*Lce1m*	66203	Late cornified envelope 1M	
−3.83	*Lce1d*	69611	Late cornified envelope 1D	
−3.79	*Lce1a2*	73722	Late cornified envelope 1A2	
−3.73	*Lce6a*	78382	Late cornified envelope 6A	
−3.56	*Lce1f*	67828	Late cornified envelope 1F	
−3.51	*Lce1e*	68694	Late cornified envelope 1E	
−3.40	*Lce1b*	68720	Late cornified envelope 1B	
−3.37	*Kprp*	433619	Keratinocyte proline-rich protein	
−3.23	*Lce1l*	73730	Late cornified envelope 1L	
−2.79	*Lce1c*	73719	Late cornified envelope 1C	
−2.48	*Lce1a1*	67127	Late cornified envelope 1A1	
−2.29	*Lce1h*		Late cornified envelope 1H	
−2.16	*Lce1j*		Late cornified envelope 1J	
−2.16	*Lce1g*	66195	Late cornified envelope 1G	
−2.67	*Lor*	16939	Loricrin	
				Filaggrin-related proteins
−5.55	*Flg2*	229574	Filaggrin2	
−3.53	*Tchh*	99681	Trichohyalin	
−2.26	*Crnn*	381457	Cornulin	
				Proteases and protease inhibitors
−3.68	*Tmprss11f*	243083	Transmembrane protease, serine 11f	
−3.02	*Serpina12*	68054	Serine (or cysteine) peptidase inhibitor, clade A (alpha-1 antiproteinase, antitrypsin), member 12	
				Lipoxygenases
−3.67	*Alox12e*	11685	Arachidonate lipoxygenase, epidermal	
*Gene expression elevated in TAM67-positive epidermis*				
				Cutaneous keratins
+1.75	*Krt6a*	16687	Keratin 6a	
+1.68	*Krt16*	16666	Keratin 16	
+1.60	*Krt17*	16667	Keratin 17	
				Early envelope
+2.40	*Sprr2b*	20756	Small proline-rich protein 2B	
+2.14	*Tgm3*	21818	Transglutaminase type III	
+2.00	*Sprr2d*	20758	Small proline-rich protein 2D	
+1.62	*Sprr2e*		Small proline-rich protein 2E	
				Proteases and protease inhibitors
+4.19	*Serpinb3a*	20248	Serine (or cysteine) peptidase inhibitor, clade B (ovalbumin), member 3A	
+2.51	*Serpinb3d*	394252	Serine (or cysteine) peptidase inhibitor, clade B (ovalbumin), member 3D	
+1.80	*Serpinb6c*		Serine (or cysteine) peptidase inhibitor, clade B (ovalbumin), member 6C	
+2.07	*Spink12*	78242	Serine peptidase inhibitor, Kazal type 12	
+2.04	*Klk13*	626834	Kallikrein related-peptidase 13	
+1.89	*Serpinb2*	18788	Plasminogen activator inhibitor 2	
+1.84	*Capn2*	12334	Calpain 2	
				Envelope defense
+3.82	*S100a8*	20201	S100A8	
+3.72	*Defb3*	27358	Defensin beta 3	
+3.19	*S100a9*	20202	S100A9	
+2.00	*Defb4*	56519	Defensin beta 4	
				Chitinase-like proteins
+4.18	*Chi3l4*	104183	Chitinase-3-like protein 3	
				Desmosome
+1.99	*Dsc2*	13506	Desmocollin 2	
+1.91	*Dsg3*		Desmoglein 3	

Larger negative or positive numbers indicate greater enrichment

a Negative log2 values indicate relative enrichment in TAM67-negative epidermis and positive numbers indicate relative enrichment in TAM67-positive epidermis

## Abbreviations

CE: cornified envelope
Krt: keratin
AP1: activator protein 1
SDS: sodium dodecyl sulfate
ARCI: autosomal recessive congenital ichthyosis
BrdU: bromodeoxyuridine
DSC: desmocollin
DSG: desmoglein
